# Screening of Forestry Workers in Guadalajara Province (Spain) for Antibodies to *Lymphocytic Choriomeningitis* Virus, Hantavirus, *Rickettsia* spp. and *Borrelia burgdorferi*

**DOI:** 10.3390/ijerph16224500

**Published:** 2019-11-15

**Authors:** Lourdes Lledó, Consuelo Giménez-Pardo, María Isabel Gegúndez

**Affiliations:** Departamento de Biomedicina y Biotecnología, Universidad de Alcalá, 28801 Alcalá de Henares, Spain; consuelo.gimenez@uah.es (C.G.-P.); isabel.gegundez@uah.es (M.I.G.)

**Keywords:** epidemiology, forestry workers, zoonosis, *Lymphocytic choriomeningitis* virus, hantavirus, *Rickettsia*, *Borrelia burgdorferi*

## Abstract

Exposure to *Lymphocytic choriomeningitis* virus (LCMV), hantaviruses, *Rickettsia* spp. and *Borrelia burgdorferi* among forestry workers from a province in central Spain (Guadalajara) was examined by serological screening. This is the first such study in this rural area, where people often live and work in proximity to domestic and wild animals. Immunofluorescent analyses of the serum of 100 forestry workers detected IgG antibodies to LCMV in 2% (CL 95% 0.55%–7.0%) of this population, to hantaviruses in 4% (CL 95% 1.6%–8.3%) for the serum amyloid A (SAA) serotype, and 2% (CL 95% 0.55%–7.0%) for the Seoul virus (SEO) serotype (samples also positive for SAA), to *Rickettsia* in 8% (CL 95% 4.1%–15%) (3% (CL 95% 1.0%–8.5%) for *R. typhi* and 5% (CL 95% 2.2%–11.2%) for *R. slovaca*, and to *B. burgdorferi* in 7% (CL 95% 3.4%–13.8%). The number of people who have been exposed to these organisms is commonly underestimated since most infections are asymptomatic. Greater epidemiological surveillance may therefore be recommended.

## 1. Introduction

Wild and domestic animals act as reservoirs for many infectious agents that can be transmitted to humans. These agents include viruses, such as *Lymphocytic choriomeningitis* virus (LCMV; family *Arenaviridae*) or hantaviruses that are transmitted to humans mainly through inhalation of aerosolized rodent excreta (feces and urine) and bacteria such as *Borrelia* spp. or *Rickettsia* spp., which are transmitted to humans through the bites of ticks or flees. People living in rural areas, especially those whose work brings them into contact with animals (like farmers or forestry workers) may be at increased risk of infection [1]. 

LCMV can cause viral meningitis in humans [2]. Rodent-borne arenaviruses are an emerging public health concern, and their circulation among the human population is commonly underestimated since most infections are asymptomatic. In Spain these viruses have received some attention, since they have been linked to neurological illness in immunocompetent persons [3].

Hantaviruses (family *Bunyaviridae)* are the etiological agents of different clinical syndromes, like hemorrhagic fever [4,5]. Although rodents were thought to be the main disease reservoir, recent studies from Côte d’Ivore and Gabon have provided evidence of shrew-borne hantavirus infections in humans [6]. It has been indicated that, in Spain, there is more than one genotype of hantavirus [7]. Certainly, hantaviruses have long been present in the Spanish regions of Catalonia [5] and Madrid (center) [7], as well as in the more northerly province of Soria [4].

In Spain, emerging species of rickettsia (family *Rickettsiaceae*), have been detected, including members of the spotted fever group (SFGR) such as *R. slovaca*, an agent of Tick-borne lymphadenopathy (TIBOLA) disease, and *R. felis* [8,9,10,11]. *R. typhi* is also a reemerging threat [12,13,14]. Rodents appear to be an important reservoir of *R. typhi*, and two transmission cycles have been described: the classic rat-flea-rat cycle and the peridomestic animal cycle in which the reservoir may be cats or dogs [14]. Certainly, *R. slovaca* appears to have two transmission cycles in which the reservoirs are either ungulates and wild boars or domestic ruminants such as sheep, goats, and cattle [9]. Antibodies to *R. typhi* and *R. slovaca* in Spanish red foxes might provide a useful indicator of the presence of these pathogens [15]. 

Finally, *Borrelia burgdorferi* (family *Spirochaetaceae*)—the cause of Lyme disease, a wildlife-involving zoonotic disease of the Northern Hemisphere [16]—has been detected serologically in northern [17,18], central [19], and southern Spain [20]. Dogs are the most important reservoirs and may be considered sentinel animals [21], although other species may also be involved in transmission [15]. People with antibodies to *B. burgdorferi* are not uncommon in Spain, but most are asymptomatic. Indeed, a study covering the period 2006–2016 in the healthcare area of Santiago de Compostela (Galicia, Spain) [22] and a seventeen-year epidemiological surveillance study in two provinces of northern Spain [23], found Lyme disease to be rare.

The present paper examines the seroprevalence of a number of potential disease-causing agents—the transmission under natural conditions of which is poorly understood—among forestry workers in a rural area of Spain. This work is the first step of a broader epidemiological study that will allow us to better understand the epidemiology of the studied infections and their importance in the study area.

## 2. Materials and Methods 

### 2.1. Study Area

**Figure 1:**
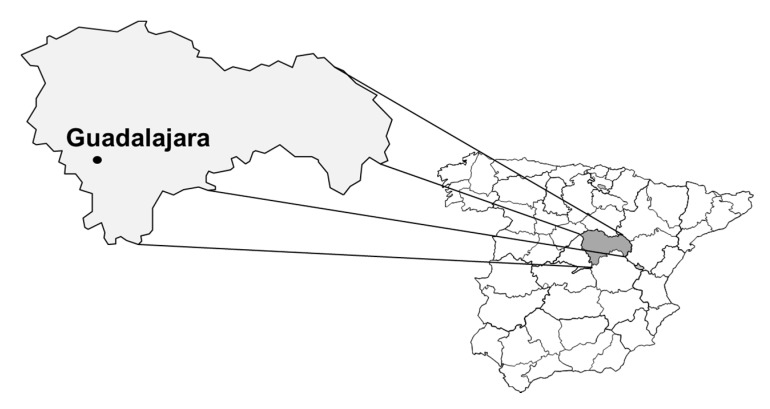
Guadalajara province.

This study was performed in Guadalajara province (located in the center of Spain, as seen in Figure 1), an area with medium sized urban populations and numerous isolated villages whose main activities are forestry, agriculture, and cattle raising.

### 2.2. Serum Samples

One hundred serum samples were obtained from forestry workers from Guadalajara province (70 men, 30 women; median age 33 years (IQR 29.5–40.25 years) and stored at −20 ºC (celsius) until analysis. The following information was recorded for each person: age, sex, contact with domestic and wild animals, and history of bite by arthropods. All subjects lived in rural areas.

The number of subjects included in the study accounted was for 95% of the province’s total forestry workers (the other 5% did not want to participate in the study). 

All subjects gave their informed consent for inclusion before they participated in the study. The study was conducted in accordance with the Declaration of Helsinki of 1975 (as revised in 2013), and the protocol was approved by the Ethics Committee of the Universidad de Alcalá (Protocol number CEI 2011034). 

### 2.3. Immunofluorescence

LCMV antibodies were detected using spot slides of L-929 cells (ATCC-CCL 1; American Type Culture Collection, Rockville, MD, USA) and infected with the Armstrong strain to provide antigens [24]. Antibodies to hantaviruses were detected using SAAV (Saaremaa strain) and Seoul (strain 80/39) viruses propagated in Vero E6 cells (ATCC-CRL 1586) and fixed on spot slides [25]. Antibodies to *Rickettsia* were detected using Vero E6 cells (ATCC 1586) infected with *R. typhi* (Wilmington strain) or *R. slovaca* (strain 246 CDC) [26]. *B. burgdorferi* antibodies were detected employing *B. burgdorferi sensu stricto* (strain B31 ATCC 35210), propagated in Barbour Kelly medium and fixed on spot slides, as an antigen source [27]. 

Fluorescein-labeled rabbit anti-human IgG antibodies were used to reveal the binding antibodies (Sigma; St. Louis, MO, USA). Positive and negative control sera were also examined. Sera showing a typical pattern of fluorescence and an IgG titer of ≥1/16 for LCMV, ≥1/32 for hantaviruses, ≥1/64 for *Rickettsia*, and ≥1/256 for *B. burgdorferi* were deemed positive. All positive samples were serially diluted to determine the endpoint titer (expressed as the reciprocal of the serum dilution). 

### 2.4. Statistical Analysis

Proportions and 95% confidence intervals were used using Wilson’s exact test. Odds ratios and their confidence intervals were estimated as a measure of risk. For the comparison of proportions, the χ^2^ test was calculated (and the Fisher’s exact test as needed). To compare mean differences, *t*-student parametric test or the Mann–Whitney nonparametric test were used. Significance was set at *p* < 0.05. 

## 3. Results

Twenty (CL 95% 13.3%–28.9%) of the women’s samples (4/30, CL 95% 5.3%–29.7%) and 16/70 (CL 95% 14.6–34%) of the men’s samples returned a positive result for at least one of the pathogens. The average age of the seropositive subjects was 41 years (IQR 31–48.25). 

IgG antibodies against LCMV were found in two serum samples (overall seroprevalence 2%, women 0% (CL 95% 0.0%–11.35%), men 2.9% (CL 95% 0.8%–9.8%)), one from a man aged 45 and the other from a man aged 54 years.

IgG antibodies against hantaviruses were found in four serum samples. The overall seroprevalence was 4% (CL 95% 1.6%–8.3%, women 3.33% CL 95% 0.6%–16.7% (1/30), men 4.3% CL 95% 1.5%–11.9% (3/70)). The average age of the seropositive subjects was 29 years (IQR 28.5–43).The total seroprevalence for the SAA serotype was 4% (CL 95% 1.6%–8.3%). Of them, 3.33% of women tested positively (CL 95% 0.6%–16.7%, 1/30) and 4.3% of men (CL 95% 1.5%–11.9% (3/70)). For the SEO serotype it was 2% seroprevalence (CL 95% 0.55%–7.0%) with women tested at 3.33% (CL 95% 0.6%–16.7% (1/30)) and men at 1.42% (CL 95% 0.3%–7.7% (1/70)). Two samples had antibodies against both the hantaviruses antigens used; in both cases, the highest titer was for the SAA virus.

IgG antibodies against *Rickettsia* were found in eight serum samples (total seroprevalence 8% (CL 95% 4.1%–15%): 3.3% of the women (CL 95% 0.6%–16.7% (1/30)) and 10% of the men (CL 95% 4.9%–19.2% (7/70)). The median age of the seropositive subjects was 29 years (IQR 28.5–43). The total seroprevalence for the *R. typhi* serotype was 3% (CL 95% 1.0%–8.5%), with 0% of women (CL 95% 0.0%–11.35% (0/30)) and 4.3% of men (CL 95% 1.5%–11.9% (3/70)). For *R. slovaca* the seropervalence was 5% (CL 95% 2.2%–11.2%) which included 3.33% of women (CL 95% 0.6%–16.7% (1/30)) and 5.71% of men (CL 95% 2.2%–13.8% (4/70)). One male sample was positive for antibodies against both *R. typhi* and *R. slovaca*. 

IgG antibodies against *B. burgdorferi* were found in seven samples (overall seroprevalence 7% CL 95% 3.4%–13.8%). 6.7% of women (CL 95% 1.8–21.3% (2/30)) and 7.14% men (CL 95% 3.1–15.6% (5/70)). The median age of the seropositive subjects was 40.5 years (IQR 32.5–48.5). One sample from a man aged 40 years was positive for *B. burgdorferi* and *R. slovaca*. 

Sixteen men (22.9%) had some seropositive serology compared to four (13%) of women (OR 1.93 CL 95% 0.59–6.34; *p* = 0.414). Seropositive people were older than seronegative (mean differences 6.5 CL 95%: 1.8–11.2 years; *p* = 0.006 (Mann–Whitney)). The difference in seropositivity between the men and women and the mean age of individuals positive for the different infectious agents are shown in Table 1. No significant differences in seroprevalence were seen with respect to the infectious agent (*p* = 0.165). 

All seropositive and seronegative subjects had contact with animals (domestic cats and dogs and wild animals, mainly rodents and foxes). All subjects, seropositive and seronegative subjects, referred a history of arthropod bites.

Table 2 shows the titers recorded for positive sera. The highest titers were recorded for *R. slovaca* and *B. burgdorferi.*

## 4. Discussion

The present study is only the first stage of a project to determine the distribution and seroprevalence of human infection with *Lymphocytic choriomeningitis* virus, hantavirus, *Rickettsia* spp., and *Borrelia burgdorferi* in Guadalajara province. The present results should therefore be considered to be only preliminary. The antigen used in the IFAT was not species-specific, since antibodies to other members of the families studied can cross-react with antigens used. In future studies, more antigens will be used in an attempt to see exactly which members of each of the genera and species studied are circulating and we will expand the study to more groups of the population of Guadalajara.

The data obtained about antibodies seroprevalence for LCMV are similar to the values that have been reported for the general population in the center of Spain (1.7%) [24] and from the south of country (1.3%) [3]. Lledó et al. (2003) reported the mean age of seropositive individuals to be 44 years, similar to the 49.5 years recorded in the present study [24]. The seroprevalence reported for the rural population in that earlier work (2.3%) was also similar to that seen in the present work (2%). The highest seropositive rate in the former study was seen in males (2.85%), all of whom worked in agricultural and stock-raising settings. LCMV infection in women is of particular interest, however, since this virus is a teratogenic pathogen [28]. It may be recommended to check for LCMV when patients present with aseptic meningitis.

The seroprevalence for LCMV reported here is similar to that recorded in other seroepidemiological studies in other areas of the world: 2.38% in Argentina [29], 5.1% in Canada [30], and 3.5% in the USA [31]. However, a study performed in different parts of North America involving people with occupations that entailed close contact with rodents, found no positive serum samples among the 757 examined [32].

While LCMV infection is known to be widespread among rodents in Spain [33], its transmission to humans under natural conditions is poorly understood [24]. Increased epidemiological vigilance may provide some answers. In any event, it would appear to be warranted: it has been recently reported that the seroprevalence of antibodies against LCMV in the Province of Trento (Italy) has been increasing over time [34].

The overall seroprevalence for hantavirus infection was 4% (CL95% 1.6%–8.3%). We used two different hantavirus antigens in two IFA tests—an assay which is sensitive and highly cross-reactive for various hantavirus antibodies—and the risk of missing any hantavirus-reactive samples was low. The present results confirm that there is more than one serotype circulating; the overall seroprevalence for SAA was 4%, and for the Seoul strain, it was 2% (this latest result may be a false positive due to cross reactions, because the two subjects were also positive to SAA to higher titers). Similar values were obtained by Sanfeliú et al. in Catalonia [5].

In a study involving persons whose occupations entailed close physical contact with rodents in North America [32], the authors concluded that the risk of being infected by hantaviruses or arenaviruses was usually low. After the existence of hantavirus infection was confirmed in Spain [4], Lledó et al. [35] reported the Seoul virus to be the more seroprevalent in biologists that had contact with rodents. It is generally believed that hantavirus infection in Spain is rare and limited to rural areas, but immunofluorescence and Western blot studies have detected seropositive persons living in urban Madrid (overall seroprevalence 0.30%) [36]. Seroprevalence among the present forestry workers was higher (4% CL 95% 1.6%–8.3%) but still very similar to rates reported for healthy populations in the Provinces of Soria [4], Madrid [7], and Catalonia [5] (overall 2%). 

Also to test the rickettsial study prevalence, we used two different antigens (*R. slovaca* and *R. typhi)* in two IFA tests, to cross-react with other *Rickettsia* (spotted fevers-group and typhus-group). Chai et al. [37] studied the seroprevalence of *R. typhi* (2.07%) in different rural areas of Zhejiang province, China and reported it to increase with subject age. Other authors have reported seroprevalence for *Rickettsia* spp. to be high in people working on farms in Brazil [38]. Podsiadly et al. [39] also reported a high seroprevalence (14.7%) for *R. conorii* among forest workers. In Catalonia (Spain), a seroprevalence of 5.5% has been reported for *R. slovaca* [40] and 8.8% for *R. typhi* [8]. Lledó et al. [41], reported a mean seroprevalence of 6.8% for the Province of Madrid. A study performed in the northern Spanish Provinces of Palencia and Burgos recorded a mean seroprevalence for *R. typhi* of 7.5%, although higher values (>12%) were detected in rural areas, with farmers and stock-breeders being the populations most at risk [13]. Bolaños-Rivero et al. [11] reported a seroprevalence of 3.9% for *R. typhi* in the Canary Islands, with no differences in terms of age and sex. The seroprevalence of 5% recorded for *R. slovaca* in the present work is similar to the 5.5% reported by Antón et al. [40] for the northeast of Spain. The presence of this bacterium in rural areas, plus its detection in Catalonian goats [42], suggest that domestic ruminants are exposed to this pathogen. A cycle in domestic animals could increase the risk of the pathogen being transmitted to humans.

Little is known about *Borrelia burgdorferi* infection, even among populations ostensibly at high risk. However, while a long-term study in two regions of northern Spain found no significant differences between occupations in terms of the seroprevalence of this organism [23], work from Poland does report differences in this respect [1,43]. The 5% CL 95% 2.2%–11.2% seroprevalence detected in the present work is low compared to that reported in the long-term study undertaken by Lledó et al. [23], but other studies performed in Madrid [19] and Navarra [44] reported similar figures of 3.45% and 4.4% respectively. In the present work, no statistical differences in seroprevalence were seen with respect to sex, unlike that seen in earlier work [19].

Finally, it is important to highlight the limitations of this pilot study: the small size of the sample analyzed (offers very large IC95%) and the low power of the study may be the reason that some of the differences found are not statistically significant. In addition, few variables (sex, age, contact with domestic and wild animals and history of bite by arthropods) were analyzed and it would be very important to increase information of other risk factors at home related to the diseases.

## 5. Conclusions

This study is the first to detect antibodies against the studied infectious agents in people—workers with relations with animals and arthropods—from this area of Spain. Although transmission of the present infectious agents to humans is low, probably, the prevalence of infection is underestimated given that most people remain asymptomatic. In summary, it is important to increase awareness for the studied infection, and better surveillance should be implemented. Further epidemiological studies are planned (as studies in the general population and in patients with signs and symptoms suggestive of being produced by these microorganisms), to increase the knowledge of the true significance of these infections in public health, because maintaining epidemiological vigilance is essential.

## Figures and Tables

**Table 1:** Data from the statistical analysis of the results.

Microorganisms	Men (*n*)	Women (*n*)	*p* Value *	OR (CL 95%)	Age (Mean)	*p* Value **
*Lymphocytic choriomeningitis* viruses (LCMV) +	2	0	1	NA	50	0.037
LCMV −	68	30			35.1	
Hantavirus +	3	1	1	1.3 (0.13–13.01)	33.5	0.438
Hantavirus −	67	29			35.5	
*Rickettsia* +	7	1	0.429	3.2 (0.38–27.41)	42.6	0.011
*Rickettsia* −	63	29			34.8	
*B. burgdorferi* +	5	2	1	1.1 (0.20–5.89)	39.6	0.157
*B. burgdorferi* −	65	28			35.1	

* Fisher exact test. ** U Mann–Whitney-bilateral-test. NA: Not applicable.

**Table 2:** Serum titers (IgG) for the studied microorganisms.

Positives	*Lymphocytic choriomeningitis* Viruses (LCMV)	Hantaviruses		*Rickettsia*		*B. burgdorferi*
		SAA	Seoul	*R. typhi*	*R. slovaca*	
1				1/64	1/64	
2		1/64	1/32			
3						1/2048
4					1/256	
5					1/512	
6					1/256	
7		1/32				
8						1/512
9				1/64		
10				1/128		
11						1/512
12	1/32					
13					1/64	1/512
14						1/256
15	1/32					
16						1/512
17		1/32				
18						1/256
19					1/64	
20		1/64	1/32			
